# Chromosomal aberrations and aneuploidy in oral potentially malignant lesions: distinctive features for tongue

**DOI:** 10.1186/1471-2407-11-445

**Published:** 2011-10-13

**Authors:** Patrizio Castagnola, Davide Malacarne, Paola Scaruffi, Massimo Maffei, Alessandra Donadini, Emanuela Di Nallo, Simona Coco, Gian Paolo Tonini, Monica Pentenero, Sergio Gandolfo, Walter Giaretti

**Affiliations:** 1 Department of Diagnostic Oncology, Biophysics and Cytometry, National Institute for Cancer Research, Genoa, Italy; 2 Department of Diagnostic Oncology, Translational Oncopathology, National Institute for Cancer Research, Genoa, Italy; 3 Department of Clinical and Biological Sciences, Oral Medicine and Oral Oncology Section, University of Turin, Italy; 4 Center of Physiopathology of Human Reproduction, Dept. Obstetrics and Gynecology, "San Martino" Hospital, Genoa, Italy

## Abstract

**Background:**

The mucosae of the oral cavity are different at the histological level but appear all equally exposed to common genotoxic agents. As a result of this exposure, changes in the mucosal epithelia may develop giving rise to Oral Potentially Malignant Lesions (OPMLs), which with time may in turn progress to Oral Squamous Cell Carcinomas (OSCCs). Therefore, much effort should be devoted to identify features able to predict the likeliness of progression associated with an OPML. Such features may be helpful in assisting the clinician to establish both appropriate therapies and follow-up schedules. Here, we report a pilot study that compared the occurrence of DNA aneuploidy and chromosomal copy number aberrations (CNAs) in the OPMLs from different oral anatomical subsites.

**Methods:**

Samples from histologically diagnosed OPMLs were processed for high resolution DNA flow cytometry (hr DNA-FCM) in order to determine the relative DNA content expressed by the DNA index (DI). Additionally, array-Comparative Genomic Hybridization (a-CGH) analysis was performed on DNA obtained from diploid nuclei suspensions directly. When aneuploid nuclei were detected, these were physically separated from diploid nuclei on the base of their DI values by means of a DNA-FCM-Sorter in order to improve the a-CGH analysis.

**Results:**

Tongue OPMLs were more frequently associated with DNA aneuploidy and CNAs than OPMLs arising from all the other mucosal subsites.

**Conclusions:**

We suggest that the follow-up and the management of the patients with tongue OPMLs should receive a distinctive special attention. Clearly, this hypothesis should be validated in a prospective clinical study.

## Background

Oral mucosae are exposed to genotoxic insults principally from chemical components contained in cigarette smoke, tobacco, food and beverages. About two thirds of oral squamous cell carcinomas (OSCCs) may be explained by tobacco cigarette and alcohol consumption [1]. In particular, tobacco smoke and alcohol appear to synergistically influence the development of oral epithelial dysplasia [2]. The role of other agents such as fungi and viruses is also recognized including the sexually transmitted human papilloma virus (HPV). This virus, in particular, appears to be linked to a recent surge of cancers especially in young individuals and in anatomical subsites located at the back of the oral cavity as well as in the pharynx [3,4]. As a result of these genotoxic insults, the normal epithelia may undergo with time to morphological changes leading to lesions appearing as patches/plaques with white (leukoplakia) or red (erythroplakia) or mixed color (erythroleukoplakia) [5]. Altogether, these lesions are referred to as Oral Potentially Malignant Lesions (OPMLs). The actual transformation rate associated with these lesions is quite different, being those with a mixed appearance at a higher risk of neoplastic progression [6]. These lesions may or may not be dysplastic. Both macroscopic and microscopic features may be evaluated differently due to the subjective criteria of classification. It is widely accepted that OPMLs associated with dysplasia deserve a closer clinical follow-up and earlier surgical treatment on the basis of a higher potential of malignant transformation with respect to non dysplastic OPMLs [7].

Concerning the anatomical subsites of development of OPMLs, it should be considered that the stratified epithelia covering the structures present in the oral cavity have diverse histological features, which may result in different levels of permeability. In addition, different oral subsites with the same pattern of epithelial differentiation were found to display different permeability properties [8]. A different exposure to genotoxic insults may also occur at particular regions due to other causes i.e., due to a pooling effect. Both different permeability and different exposure may be reflected at the pathophysiological level by different rates of occurrence of OPMLs and progression of these lesions to malignant transformation among different subsites.

Most OSCCs arise from the floor of the mouth, the soft palate complex and the lateral/ventral tongue [9,10]. Concerning the tongue, oncologically relevant lesions involve almost exclusively its lateral and ventral subsites, sparing the dorsum. It has also been reported that the lateral tongue displays a high recurrence rate after surgery for OSCC [11]. Interestingly, however, a large study showed that there was no survival difference between patients with tongue cancer and cancers located at other oral cavity anatomical subsites [12].

It is conceivable that the epithelia covering subsites at high risk of cancerization [13] are more frequently associated with dysplasia. Alternatively, dysplastic lesions at these subsites may have a higher propensity to undergo malignant transformation.

Whatever hypothesis is true, it is expected that large genomic changes such as DNA aneuploidy, should be found more frequently associated with OPMLs arising from subsites at high risk of cancerization than in low risk subsites. Along this line, an early report indicated that DNA aneuploidy proved to be a prognostic indicator of malignancy progression in oral cancer [14]. Recently, it has also been proposed that DNA ploidy analysis helps to identify oral epithelial dysplasia with a high risk of malignant progression [15]. Furthermore, a pilot study reported that dysplasia from high risk subsites have a greater tendency to include genetic aberrations such as loss of heterozygosity (LOH) on 3p and/or 9p associated with elevated risk of progression [16].

In the present study, we took advantage of high resolution DNA flow cytometry (hr DNA-FCM) to detect subclones with DNA aneuploidy. Additionally, we evaluated by a-CGH chromosomal segmental gains and losses and chromosomal numerical aberrations, which are generally defined as DNA copy number aberrations (CNAs). This last approach was done, for the DNA aneuploid lesions, after the sorting DNA aneuploid epithelial nuclei. Using these techniques, we aimed to explore the relationship among OPML anatomical subsite, DNA aneuploidy and CNAs.

## Methods

### Patients and lesions

Patients with OPMLs were recruited in two different medical centers: the Oral Medicine and Oral Oncology Section of the University of Turin at the A.S.O. S. Luigi Gonzaga (Orbassano-Turin) and at the Department of Otolaryngology, "S. Martino Hospital" in Genoa. Patient written consent was obtained in every case according to the Institutional Ethic Committees (A.S.O. S. Luigi Gonzaga Prot. N. 11780 and S. Martino Hospital Prot. N. 1084) during an interview in which their tobacco and alcohol habits were recorded.

Inclusion criteria were the presence of one or more OPMLs (homogeneous and non-homogeneous leukoplakias, erythroplakias and erythroleukoplakias) affecting only a single oral cavity subsite and not extending to other subsites. The patients were all at first presentation, with no history of a previous cancer of the oral cavity.

For each individual OPML, micro-biopsies (by means of a curette) and incisional biopsies were obtained as previously detailed [17]. In performing the micro-biopsies, care was taken to cause slight bleeding to ensure that the basal layers of the epithelium had been collected [17]. The samples from the OPMLs, when these were relatively large, included also the lesion margins. Micro-biopsies were also obtained from "oral distant fields" (ODFs) at a distance from co-existing OPMLs and characterized by visually normal appearing mucosa.

Both micro-biopsies and incisional biopsies were subdivided in order to serve for both FCM and a-CGH analyses and histological diagnosis. The samples for DNA-FCM and a-CGH were either immediately submitted to FCM analysis or stored at -20°C to be processed at a later time. The histological diagnosis of dysplasia in ODFs and OPMLs was carried out by a specially trained pathologist, according to the WHO guidelines [17,18]. ODF samples never harbored dysplasia. Dysplastic lesions included all grades of dysplasia.

Specific data concerning lesion size and homogeneity could not be reliably retrieved from the patients' clinical files.

Table 1 reports the number of patients enrolled in the study and the type and number of tissue samples used to perform FCM and a-CGH analyses. Some patients presented multiple lesions in the same anatomical subsite. Patients with tissue samples derived from the tongue or from all the other subsites were referred to as T or O patients respectively.

**Table 1:** Patients enrolled in the study, type and number of samples used to perform the FCM and aCGH analyses.

	FCM analysis	
	Tissue Samples	Patients
	
ODFs	70	
		107^§^
OPMLs	172	
		
	**a-CGH analysis**	

	Tissue Samples	Patients
	
OPMLs	35	27*

^§^Fifty-six males and 51 females, with a median age of 58 years, range 26-86 without actual or previous oropharyngeal cancer. Sixty-four patients showed presence of lesions on the buccal mucosa, 21 on the tongue, 8 on the gum and 14 on other subsites.

*These 13 male and 14 female patients, with a median age of 64, range 26-83, were a subgroup of the 107 patients examined by FCM analysis. Fourteen patients showed presence of lesions on the buccal mucosa, 8 on the tongue, 3 on the gum and 2 on other subsites.

### Processing of tissue samples for high resolution DNA flow cytometry (hr DNA-FCM) analysis and sorting

Tissue samples were processed to obtain DAPI stained nuclei suspensions following the method of Otto et al. [19] with modifications. High resolution DNA flow cytometry (hr DNA-FCM) of these samples was used as previously described in details[20] to obtain DNA content histograms and to evaluate the DNA Index (DI). DNA diploid controls were represented by gender-specific human lymphocytes. The mean CV of the corresponding DNA diploid GO-G1 peaks was 1.2 ± 0.2%. The DNA diploid (DI = 1) and aneuploid sublines (DI≠1) detected in ODFs and OPMLs were sorted using a Cyflow Space FCM equipped with a PPCS unit (Partec GmbH, Muenster, Germany) at the purity of about 99%. Patients with tissue samples derived from the tongue or from all the other subsites were referred as T patients or O patients respectively. DI values were determined in 21 T and 86 O patients (Table 2).

**Table 2:** Distribution of patients with OPMLs+ODFs in the tongue (T patients) or in all the other oral mucosal regions (O patients) or in Buccal Mucosa (BM patients) with respect to DNA diploid or aneuploid status.

		OPMLs+ODFs	
	**O patients vs. T patients**	(OR = 6.1; *p *= 0.0008)*	
		
		**T patients vs. BM patients**	**(OR = 7.0; *p *= 0.001)**^**§**^
	
	**O patients**	**T patients**	**BM patients**

DNA diploid	68	8	52
DNA aneuploid	18 (21%)	13 (62%)	12 (10%)

Total	86	21	64

*Once stratified for presence/absence of dysplasia in the OPMLs, OR_M-H _= 5.5 (p = 0.002).

^§^Once stratified for presence/absence of dysplasia in the OPMLs, OR_M-H _= 5.9 (p = 0.001).

The DNA aneuploid sublines detected by hr-DNA FCM were physically separated from the DNA diploid components with a FCM-sorter and submitted, as a pure subpopulation of nuclei from epithelial cells, to a-CGH to provide a genome-wide measurement of DNA copy number aberrations (CNAs).

Concerning DNA diploid lesions, no enrichment for epithelial cell nuclei was performed. Therefore, diploid nuclei from non-epithelial cells may be present in the sample processed for a-CGH.

After DNA FCM analyses and before DNA extraction, both aneuploid and diploid nuclei were washed twice with H_2_O at 3000 rpm, for 15 minutes at 10°C.

### High resolution a-CGH analysis

The measurements of CNAs were performed on 6 DNA aneuploid and 29 DNA diploid samples from 27 patients (8 T and 19 O patients, respectively) using the high resolution oligonucleotide Human Genome CGH 105 K array platform (Agilent Technologies, Santa Clara, CA). DNA was extracted using ArchivePure DNA kit (5-Prime Hamburg, Germany) with some modifications: after proteins precipitation the DNA was purified by phenol-chloroform extraction and collected by ethanol precipitation adding 20 μg of glycogen (20 mg/ml) as carrier. DNA quality and quantity were assessed by agarose gel and ND-1000 spectrophotometer (Thermo Scientific, Wilmington, DE). A pool of normal male or female DNA (Promega, Madison, WI) was used as reference DNA. A quantity of 50 ng of genomic DNAs (test and reference) was amplified by GenomePlex Whole Genome Amplification Kit (Sigma-Aldrich, St. Louis, MO) that allows to generate a representative amplification of genomic DNA. The kit uses a linker mediated primer PCR amplification technology based upon random fragmentation of genomic DNA and conversion of the resulting small fragments to PCR-amplifiable OmniPlex Library molecules flanked by universal priming sites. The OmniPlex library is then PCR amplified using universal oligonucleotide primers. After purification of PCR products by GenElute PCR Clean-Up Kit (Sigma-Aldrich), amplified DNA was quantified using the ND-1000 Spectrophotometer (Thermo Scientific). For each array, 2 μg amplified test and reference DNAs were labeled by Bioprime Labeling Kit (Invitrogen, Paisley, UK) following the manufacture's protocol. Unincorporated nucleotides were removed on Microcon YM-30 filters (Millipore, Billerica, MA) according to the manufacturer's protocol. Labeled DNA quality analysis and quantization were performed by NanoDrop ND-1000 Spectrophotometer and parameters that predicted successful hybridization were a specific activity of 25 to 40 and 20 to 35 for cyanine-3 and cyanine-5 labeled sample, respectively. Prior to the denaturation step at 95°C for 3 min, 50 μg Cot-1 DNA (Invitrogen) and control targets (Agilent Technologies) were added to cyanine 5- and cyanine 3-labeled DNA mixture. Hybridization mix was incubated at 37°C for 30 min, and applied to array. Hybridization was carried out for 40 hr at 65°C in a rotating hybridization oven (Agilent Technologies) at 20 rpm. After washes according to the manufacture's recommendations, spot fluorescence was measured by the Agilent G2565BA Scanner and images were processed by Feature Extraction software (version 9.5.3.1) (Agilent Technologies). Data files were analyzed by Agilent CGH Analytics Software (version 3.5.14) according to ADM-2 algorithm. The quality of each experiment was assessed by QC metric tool. Raw data were uploaded in Gene Expression Omnibus (http://www.ncbi.nlm.nih.gov/geo/query/acc.cgi?acc=GSE28868)

### Relationship between DNA copy number aberrations (CNAs) and oral mucosa subsites

In order to investigate the link between CNAs and oral subsites, we first performed a contrast match with respect to sample DNA aneuploidy and with respect to smoke habit and gender of the patients. Overall, we obtained 37 different tissue samples from 27 patients (8 T patients and 19 O patients respectively) already assayed by hr DNA-FCM analysis (Table 1). Subsequently, the incidence of CNAs occurring in the samples of the two groups of patients was determined.

The sum of the segmental and numerical aberrations, which occurred within 41 chromosomal arms (no a-CGH probes were available for p arms of chromosomes 13, 14, 15, 21 and 22), irrespective of the size of the genomic region involved, were referred to as total gain, total loss and total gain+loss of genomic DNA. The sum was obtained by assigning a value of 1 for each chromosomal arm showing one or more CNAs events. In this way, each patient/lesion was characterized by a score ranging from 0 (no event detected) to 41 (gain/loss/gain+loss events detected in every arm of the chromosomes for which a-CGH probes are available). The rationale that prompted us to adopt this approach is that the pathophysiological consequences, in terms of genetic instability and risk of cancer development for the oral mucosa, of either large or small chromosomal aberrations are not fully established.

When multiple tissue samples were available from a single oral lesion, the highest sum value per chromosomal arm was assigned. By using this approach, we obtained for each patient/lesion a single aberration value.

## Results

### Tongue OPMLs have a higher occurrence of DNA aneuploidy with respect to non-tongue OPMLs

In order to evaluate the relationship between DNA aneuploidy and oral mucosa subsites, the patients were subdivided between those having tissue samples from tongue (T patients, n = 21), those having tissue samples from all the other sites (O patients, n = 86) and those having tissue samples from Buccal mucosa (BM patients, n = 64). These latter group, which was the most represented subsite, was used to address whether the Tongue patients' lesions were different in terms of DNA aneuplody with respect to the lesions arising in a different specific oral mucosa subsite. The incidence of DNA aneuploidy occurring in the samples of the three groups of patients was determined. When both DNA aneuploid and DNA diploid sublines were detected in the same tissue sample, we took as representative the aneuploid one. Table 2 shows that DNA aneuploidy was more frequent among T patients with respect to both O patients (*p *= 0.0008) and BM patients (*p *= 0.001).

To verify whether the presence of ODFs in addition to the OPMLs could affect the results, we performed two separate analyses on OPMLs (Table 3) and on ODFs (Table 4), which confirmed that DNA aneuploidy was significantly more frequent among T patients with respect to both O patients and BM patients.

**Table 3:** Distribution of patients with OPMLs in the tongue (T patients) or in all the other oral mucosal subsites (O patients) or in Buccal Mucosa (BM patients) with respect to DNA diploid or aneuploid status and to dysplastic or non-dysplastic status.

		Dysplastic + Non-dysplastic OPMLs	
	O patients vs. T patients	(OR = 5.2; *p *= 0.003)*	
		
		T patients vs. BM patients	(OR = 5.9; *p *= 0.002)^§^
		
	O patients	T patients	BM patients
DNA diploid	71	10	54
DNA aneuploid	15 (17%)	11 (52%)	10 (16%)

Total	86	21	64

		**Non-dysplastic OPMLs**	

	O patients vs. T patients	(OR = 3.6; *p *= 0.07)	
		
		T patients vs. BM patients	(OR = 3.6; *p *= 0.069)
		
	O patients	T patients	BM patients
DNA diploid	65	9	48
DNA aneuploid	12 (16%)	6 (40%)	9 (16%)

Total	77	15	57

		**Dysplastic OPMLs**	

	O patients vs. T patients	(OR = 10; *p *= 0.12)	
		
		T patients vs. BM patients	(OR = 36; *p *= 0.029)
		
	O patients	T patients	BM patients
DNA diploid	6	1	6
DNA aneuploid	3 (33%)	5 (83%)	1 (14%)

Total	9	6	7

*Once stratified for presence/absence of dysplasia in the corresponding OPMLs, OR_M-H _= 4.5 (*p *= 0.005).

^§ ^Once stratified for presence/absence of dysplasia in the corresponding OPMLs, OR_M-H _= 5.2 (*p *= 0.003).

**Table 4:** Distribution of "oral distant fields" (ODFs) collected from patients with OPMLs in the tongue (T patients) or in all the other oral mucosal regions (O patients) or in Buccal Mucosa (BM patients) with respect to DNA diploid or aneuploid status.

		ODFs	
	**O patients vs. T patients**	(OR = 11; p = 0.005)*	
		
		**T patients vs. BM patients**	**(OR = 10; p = 0.007)**^**§**^
		
	**O patients**	**T patients**	**BM patients**

DNA Diploid	53	8	48
DNA aneuploid	3 (5%)	5 (38%)	3 (6%)

Total	56	13	51

*Once stratified for presence/absence of dysplasia in the corresponding OPMLs, OR_M-H _= 14 (*p *= 0.003).

^§^Once stratified for presence/absence of dysplasia in the corresponding OPMLs, OR_M-H _= 13 (*p *= 0.004).

**Figure 1:**
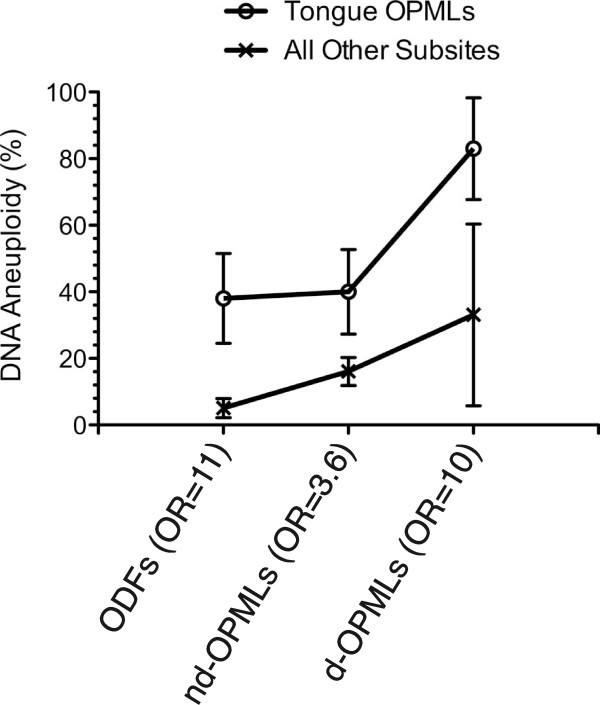
**Percentage of DNA aneuploid tissue samples, as determined by high resolution DNA flow cytometry (hr DNA-FCM), found in the tongue and in all the other anatomical oral mucosa subsites for Oral Distant Fields (ODFs), non-dysplastic and dysplastic Oral Potentially Malignant Lesions (nd-OPMLs and d-OPMLs, respectively)**. OR, odd ratio. The numerical data are shown in Tables 3 and 4. Standard errors for each data point are shown as vertical bars.

Figure 1 shows the frequency of DNA aneuploidy among ODFs, non-dysplastic OPMLs and dysplastic OPMLs subdivided in either the tongue or all the other anatomic subsites.

### Tongue OPMLs have a higher occurrence of CNAs with respect to OPMLs in all the other mucosa anatomical sites

We tested the hypothesis that the frequencies of CNAs in OPMLs of T patients and O patients were different. As detailed in Materials and Methods, we used a score related to CNA gains and losses. A subset of 27 patients (8 T patients and 19 O patients, respectively) were analyzed.

**Figure 2:**
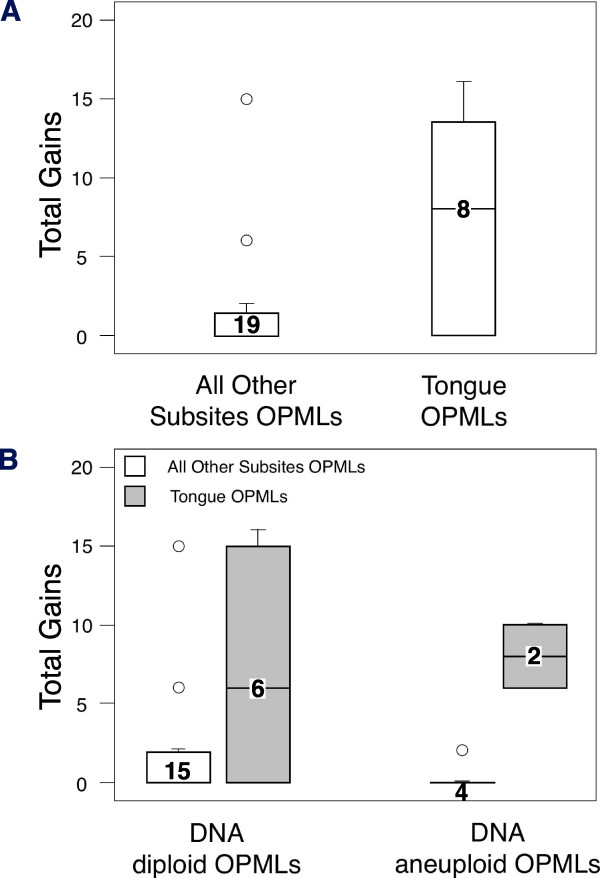
**Total gains of CNAs (see Material and Methods for details) of the tongue Oral Potentially Malignant Lesions (OPMLs) or of OPMLs from all the other anatomical mucosa subsites**. The values ranged from 0 (no DNA copy gains) to 41 (one or more gains found in each one of the investigated chromosomal arms). (A) Unstratified data. (B) Data stratified for DNA aneuploidy. The number of samples in each group is shown. Each box represented the median and the first and third quartiles, and the whiskers extended as far as the minimum and maximum values after exclusion of the outliers (values more than 1.5 interquartile ranges below the lower quartile or above the upper quartile). Excluded outliers (with value less than 3 interquartile ranges outside the first and third quartiles) were displayed as circles.

Figure 2A shows that the total gain, including both segmental and numerical aberrations (see Materials and Methods), was higher in the tongue OPMLs than in the OPMLs from all the other subsites (*p *= 0.032 ANOVA F-test; *p *= 0.085 Mann-Whitney test). A similar result was obtained when only segmental chromosomal aberrations were analyzed (*p *= 0.072 ANOVA F-test; *p *= 0.07 Mann-Whitney test; data not shown). Figure 2B shows the total gains (including both segmental and numerical aberrations) for tongue and non-tongue OPMLs after subdividing them according to DNA ploidy. Tongue DNA aneuploid OPMLs were characterized by a higher total gains than non-tongue DNA aneuploid OPMLs (*p *= 0.002 ANOVA F-test; *p *= 0.033 Mann-Whitney test). A statistically significant difference was also found when only segmental chromosomal aberrations were considered for the analysis (*p *= 0.001 ANOVA F-test; *p *= 0.028 Mann-Whitney test; data not shown). Stratification for histology grade, smoking habit or gender, maintained the observed relationship between the CNAs total gains and anatomical subsite (data not shown).

The same analyses were performed considering the total losses or the combined gains plus losses providing no statistically significant differences (data not shown).

Concerning the chromosomal arms showing statistical significant segmental gains (p < 0.05) in the Tongue OPMLs with respect to all the other oral mucosa sites, we found the 7p e 20q (present in 5 out of 8 Tongue samples), 1p, 16p e 16q (present in 4 out of 8 Tongue samples).

## Discussion

Over the last decade, oral cancer prognosis remained quite dismal if compared with the improvement achieved for cancers arising in other anatomical districts. Despite existing recommendations for early detection, this failure was most likely due to the fact that the diagnosis of oral cancer was performed at advanced stages of the disease. Therefore, a prompt recognition of OPMLs with a high potential of cancer development and the early detection of OSCC appear essential to improve patients' outcomes.

This pilot study clearly indicated that tongue OPMLs (mainly leukoplakias) were associated with a high frequency of DNA aneuploidy and CNAs with respect to OPMLs in all the other mucosa subsites. In particular, this finding held also when we compared tongue and buccal mucosa, which was the most frequently affected subsite in the present series.

The preferential development of dysplasia and DNA aneuploidy in the tongue OPMLs may suggest to the clinician to adopt a shorter time interval in the follow-up. An early large study on oral premalignant lesions indicated that, in addition to OPMLs in the floor of the mouth and in the lips, tongue leukoplakias were at a higher risk being more frequently associated with dysplasia and carcinoma [13]. Furthermore, in optimal agreement with our data, a recent pilot study demonstrated, by using image cytometry, that OPMLs originating from the tongue displayed a higher frequency of DNA aneuploidy than OPMLs from all the other anatomical subsites [21].

Remarkably, we also found that the frequency of DNA aneuploidy in the tongue ODFs with respect to the ODFs from all the other oral subsites was much higher. This may partially result from the fact that on average the distance between the tongue ODFs and the corresponding OPMLs was smaller with respect to the distance between the ODFs and OPMLs in all the other oral subsites.

Our results appear in good agreement with previously published DNA ploidy studies [13,20-22]. In addition, we added here clear evidence that tongue OPMLs displayed a higher occurrence of gain-related CNAs. This finding was clearly evident with the use of a composite biomarker (total gain of CNAs), which was simply the sum of numbers of gains from every single chromosome. We suggest that this new biomarker may be useful to assess the potential transformation risk associated to OPMLs. It may be also worth to further investigate whether specific chromosomal aberrations associated with tongue OPMLs (for example, the segmental gains in 7p, 20q, 1p, 16p and 16q) may contain gene loci with oncogenic potential.

Altogether, our data indicate that the detection of histological dysplasia, DNA aneuploidy and chromosomal aberrations in the tongue OPMLs might be associated with a higher risk of cancer development. This suggestion, however, though in agreement with previous reports specifically addressing DNA aneuploidy as a biomarker of this risk [15,23], is not yet proven. On the other hand, it is important to point out that the predictive value of both dysplasia and anatomical subsite remains in dispute [24-28]. Work is in progress to perform a prospective clinical study with an appropriate cohort of patients and a long-lasting follow up to challenge the usefulness of the presently proposed biomarker.

## Conclusions

Tongue OPMLs were more frequently associated with DNA aneuploidy and CNAs than OPMLs arising from all the other mucosal subsites. We suggest, therefore, that the follow-up and the management of the patients with tongue OPMLs should receive a distinctive special attention.

## Competing interests

The authors declare that they have no competing interests.

## Authors' contributions

PC participated in the design of the study, analyzed the data and wrote the manuscript. DM participated in the design of the study, performed the statistical analysis and analyzed the data. MM, AD and ED, performed the flow cytometry analysis and sorting and analyzed the data. PS and SC performed the CGH analysis and analyzed the data. GPT supervised the CGH analysis and analyzed the data. MP and SG were responsible for patients' interview, patients' treatment, sample collection and analyzed the data. WG conceived the study, participated in the design of the study and analyzed the data.

All authors read and approved the final manuscript.

## Pre-publication history

The pre-publication history for this paper can be accessed here:

http://www.biomedcentral.com/1471-2407/11/445/prepub
